# Machine learning for screening laryngopharyngeal reflux symptoms in college students: a cross-sectional study

**DOI:** 10.1080/07853890.2025.2610063

**Published:** 2026-01-05

**Authors:** Shuang Li, Guoji Wang, Haixian Guo, Jinzhang Cheng, Dan Yu

**Affiliations:** a Department of Otolaryngology-Head and Neck Surgery, The Second Hospital of Jilin University, Changchun, Jilin Province, P.R. China; b School of Mathematics and Statistics, Changchun University of Technology, Changchun, Jilin Province, P.R. China

**Keywords:** Dietary patterns, gastroesophageal reflux disease, Genetic Algorithm–Stacking, laryngopharyngeal reflux symptoms, lifestyle behaviours

## Abstract

**Bckground:**

Laryngopharyngeal reflux (LPR) is a widespread global health issue. Its recurring symptoms and impact on quality of life create significant economic burdens for individuals and society. To examine the links between lifestyle, diet, and LPR symptoms (LPRS) in college students, and to build an LPRS screening model using a Genetic Algorithm (GA)–Stacking method.

**Patients and Methods:**

A cross-sectional study of 502 undergraduates from 21 universities in Jilin Province, China, using an electronic questionnaire. LPRS were assessed via the Reflux Symptom Index (RSI). Associations were analyzed with multiple methods, and a GA–Stacking screening model was developed.

**Results:**

LPRS prevalence was 50.20% (252/502). Significant risk factors included frequent fried food consumption (OR: 1.89; 95% CI, 1.35-2.64), late-evening meals (OR: 2.15; 95% CI, 1.54-3.01), and low physical activity (OR: 1.72; 95% CI, 1.23-2.41). The GA–Stacking model performed well, with a recall of 0.909, accuracy of 0.927, and AUC of 0.96 (95% CI, 0.94-0.98).

**Conclusions:**

Modifiable factors like fried food intake and meal timing are strongly linked to LPRS in students. The GA–Stacking model effectively identifies high-risk individuals for early intervention, highlighting the role of lifestyle changes and informing targeted health strategies.

## KEY MESSAGES

- Lifestyle behaviours and dietary patterns demonstrated significant associations with the presence of LPRS among college students.
- A Genetic Algorithm–Stacking screening model, developed using lifestyle and dietary variables, achieved high accuracy in identifying individuals with high LPRS burdens.

## Introduction

Laryngopharyngeal reflux (LPR) is an inflammatory disorder of the upper aerodigestive tract that arises from direct or indirect exposure to gastroduodenal contents [1–3]. The most frequent laryngopharyngeal symptoms accompanied with LPR are sensation of foreign body in the throat, hemming, hoarseness and excessive mucus in the throat [4]. Since the initial identification of LPR in 1968, a progressive increase in related outpatient visits has been observed, contributing to a significant economic burden for both patients and society [5,6]. Due to the lack of a ‘gold standard’ for diagnosis, the current incidence and prevalence of LPR remain unclear. Although 24-h pH impedance monitoring can serve as an objective indicator for diagnosing LPR, false-negative results may occur with pH impedance monitoring due to the natural course characteristics of LPR [7,8]. Furthermore, the probe positioning method and diagnostic criteria have not yet been unified, making it difficult to widely apply in clinical practice and large-scale population screening.

To improve the efficiency of LPR diagnosis and screening, Belafsky et al. developed the Reflux Symptom Index (RSI) in 2001, which has now been widely used in countries including China, France and Italy [9–14]. Notably, the prevalence of LPR based on RSI scores has been reported to vary across countries, as well as across regions within China [14–16]. These geographical differences may be attributable to regional variations in lifestyle behaviours and dietary patterns. Currently, there are only a limited number of studies focusing on the impact of diet on LPR [17–19]. However, these studies have not targeted specific populations, making it difficult to comprehensively evaluate the correlations between lifestyle behaviours, dietary habits and laryngopharyngeal reflux symptoms (LPRS) in a particular group. Additionally, there is a lack of systematic sorting and analysis of lifestyle behaviours and dietary habits related to LPRS in specific populations.

Traditional statistical analyses can identify correlations of individual factors, but they struggle to integrate multiple factors to efficiently identify high-risk individuals with the complex characteristics. In contrast, machine learning models have demonstrated advantages in handling such classification problems involving multivariate and nonlinear relationships. Nevertheless, research applying these models to questionnaire-based LPRS screening remains scarce.

In summary, this study aims to explore the correlations between the self-reported lifestyle behaviours, dietary habits, and LPRS among college students through a cross-sectional survey. Based on the analysis results, a Genetic Algorithm (GA)–Stacking classification model will be developed to identify and screen individuals who currently exhibit a high risk of LPRS, with the goal of providing an efficient auxiliary tool for large-scale population preliminary screening.

## System and methods

### Questionnaire design and study participants

Ethical approval for this anonymous, web-based survey was obtained from the institutional ethics committee (2022-155). The study was conducted in accordance with the principles outlined in the Declaration of Helsinki. Informed consent was obtained from all participants prior to data collection.

A 34-item questionnaire was developed based on a comprehensive literature review and the RSI (Supplementary materials). Survey reliability was assessed using Cronbach’s α coefficient, while construct validity was evaluated using the Kaiser–Meyer–Olkin (KMO) measure of sampling adequacy and Bartlett’s test of sphericity. All analyses were conducted using SPSS (version 26 for Mac; NCSS LLC, Kaysville, UT, USA). The results indicated acceptable internal consistency reliability and good construct validity.

This cross-sectional study was conducted between October and November 2022. A multi-stage sampling design was employed to recruit a representative sample of college students from Jilin Province, China (Figure S1 and Supplement Table 1). First, a stratified random sampling method was used to select 21 universities, ensuring representation across different institutional types and sizes. Subsequently, within each participating university, quota sampling was implemented based on key demographic variables to determine the final sample distribution, thereby enhancing the sample’s representativeness and mitigating potential selection bias. The inclusion criteria were as follows: (1) willingness to participate and ability to provide informed consent, (2) ability to read and understand all questionnaire items, (3) the absence of any underlying or congenital diseases and (4) no current acute illness. The exclusion criteria were as follows: (1) individuals with specific confounding diseases (such as chronic rhinosinusitis, allergic rhinitis and asthma), (2) individuals in the acute infection phase and (3) individuals with relevant surgical history or currently receiving acid-suppressive therapy.

### Sample size justification

An a priori sample size calculation was conducted using G*Power software version 3.1.9.7 [Heinrich-Heine-Universität Düsseldorf , Germany]. Based on a pilot study and previous literature, we anticipated the medium effect size (Cohen’s f = 0.25) for the primary logistic regression analysis. With an alpha error probability of 0.05 and a statistical power of 95% (1−β = 0.95), the calculation indicated a minimum required sample size of 410 participants. Accounting for a potential non-response or data incompleteness rate of approximately 20%, we aimed to recruit at least 500 participants. Our final sample of 502 participants therefore meets and exceeds this requirement.

### Questionnaire distribution

Tutors and lecturers were initially contacted to inform students about the survey. A unique QR code, generated *via* the Questionnaire Star platform, was then distributed to invite voluntary participation. After providing informed consent, participants scanned the QR code using the WeChat app to access and complete the electronic questionnaire. The questionnaire collected information on demographic characteristics, lifestyle behaviours, dietary patterns and the presence of LPR symptoms. Due to the anonymous and web-based nature of the survey, the exact number of individuals who declined participation could not be tracked. However, stratified and quota sampling methods were employed to enhance sample representativeness.

### Evaluation of LPRS

LPRS were assessed using the RSI, which comprised nine items, each rated on a six-point scale ranging from 0 to 5. Higher total scores indicated greater symptom severity. Based on established cut-off values in the literature, participants were categorized into two groups: with LPRS (RSI > 13) and without LPRS (RSI ≤ 13) [20]. Data on symptom seasonality and duration were also collected.

### Body Mass Index

Body Mass Index (BMI) was calculated using the formula: weight (kg)/height [2] (m^2^). In accordance with the classification criteria for Chinese adults defined by the Working Group on Obesity, BMI was categorized into four groups: underweight (<18.5 kg/m^2^), normal weight (18.5–23.9 kg/m^2^), overweight (24.0–27.9 kg/m^2^) and obesity (≥28.0 kg/m^2^).

### Statistical analysis

Questionnaires with any missing data were excluded from the final analysis to ensure data completeness. Bivariate correlation analysis was conducted to examine the associations between LPRS and various features. Pearson’s correlation coefficient (PCC), maximal information coefficient (MIC), random forest (RF), CatBoost and logistic regression (LR) analysis were employed to estimate the relative contribution of lifestyle behaviours and dietary patterns. A composite feature ranking score was computed using the following formula:

$$\begin{array}{c} \text{Rank}=\omega_{1}R_{\text{PCC}}+\omega_{2}R_{\text{MIC}}+\omega_{3}R_{\text{RF}}+\omega_{4}R_{\text{CatBoost}}+\omega_{5}R_{\text{LR}}\\ \omega_{1}=\omega_{2}=\ldots =\omega_{5} \end{array}$$

The out-of-bag (OOB) error from the RF was used to identify features with the greatest predictive value for LPRS. MIC was further applied to analyse the strength of each selected feature [21]. All analyses were conducted using Python (The operating system is Windows 11 Professional Edition, with an Intel Core i7-13700KF CPU, an ROG RTX 4080 16GB GPU, and 32GB of DDR5 memory. The development environment is Python 3.8, and the compilation software is Jupyter Notebook.)

### Establishment of GA–stacking screening model

The entire dataset was randomly divided into a training set (*n* = 246), an internal test set (*n* = 106) and an independent external validation set (*n* = 150). This strict separation ensured an unbiased assessment of the final model’s performance on the external validation set. All continuous features were normalized to zero mean and unit variance before model training. A two-level stacking ensemble framework was employed. At Level-0, five base learners were implemented: Support Vector Machine (SVM) [22], Extreme Gradient Boosting (XGBoost) [23], Gradient Boosting Decision Tree (GBDT), Random Forest (RF) and K-Nearest Neighbour (KNN). The meta-learner at Level-1 was Logistic Regression (LR). To prevent target leakage and overfitting, the meta-learner was trained exclusively on out-of-fold predictions generated from the base learners during the cross-validation process on the training set. A Genetic Algorithm (GA) was utilized to optimize the hyperparameters of all learners simultaneously within the stacking framework (the optimization workflow is depicted in Figure S3) [24,25]. The GA was configured with a population size of 100, evolved over 100 generations, and employed tournament selection, single-point crossover (probability = 0.8) and mutation (probability = 0.01). The fitness of an individual in the GA population was defined as the mean Accuracy obtained from a stratified fivefold cross-validation on the training set. An early stopping rule was applied, halting the optimization if no improvement in the best fitness was observed for 20 consecutive generations. The specific hyperparameter search spaces for each algorithm, optimized by the GA, are detailed in Supplement Table S2 (e.g. C and gamma for SVM; n_estimators and max_depth for tree-based models; n_neighbors for KNN). The final GA–Stacking model with optimal parameter configuration was evaluated on the reserved independent validation set (*n* = 150) to verify its performance.

Model performance in screening LPRS was evaluated using three metrics: accuracy, recall and F1-score. These were calculated as follows:

$$\begin{array}{l} \text{Seed Vigor}\;\text{Index}\\ \;=\text{Seedling length}\;\left(\text{cm}\right)\times \;\text{Seed Germination}\left(\%\right) \end{array}$$

$$\text{Recall}=\frac{\text{TP}}{\text{TP}+\text{FN}}$$

$$\text{F1‐Score}=\frac{\text{2 }\times \text{ TP}}{\text{2 }\times \text{ TP}+\text{FN}+\text{FP}}$$

where true positive (TP) refers to the number of individuals with LPRS correctly screened as having LPRS; false positive (FP) refers to the number of individuals without LPRS incorrectly screened as having LPRS; true negative (TN) refers to the number of individuals without LPRS correctly screened as not having LPRS; and false negative (FN) refers to the number of individuals with LPRS incorrectly screened as not having LPRS.

The performance of the proposed GA–Stacking model was evaluated by comparing its accuracy, recall and F1-score against seven mainstream classifiers (SVM, XGBoost, GBDT, RF, KNN, etc.) and a baseline stacking model without GA optimization. To ensure a rigorous and fair comparison, the hyperparameters of all competing models were optimized using the same GA framework and the identical search spaces (as detailed in Supplement Table S2) applied to the GA–Stacking model. All models were subsequently trained and evaluated on the identical data splits and feature sets as described above. This approach ensures that performance differences are attributable to the model architectures and ensemble strategy, rather than disparities in parameter tuning.

## Results

### Incidence and symptom characteristics of LPRS

**Figure 1.**
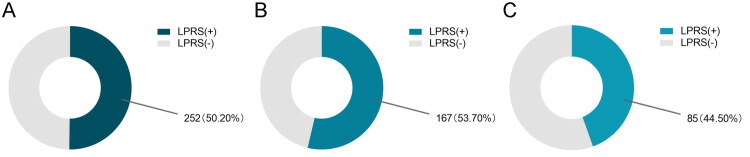
**Pie charts present the incidence of LPRS among college students.** (A) Incidence of LPRS in both sexes. (B) Incidence of LPRS in females. (C) Incidence of LPRS in males.

Among the 502 valid respondents, the overall prevalence of LPRS was 50.20% (252/502), with a higher prevalence in females (53.70%, 167/311) than in males (44.50%, 85/191) (Figure 1). Throat clearing was the most common symptom, while breathing difficulties and choking episodes were relatively rare (Table 1). The RSI score was positively correlated with individual LPRS, with the strongest correlation observed with the sensation of a foreign body in the throat (S8) (Table 2). In addition, significant correlations existed among multiple symptoms (e.g. postprandial/recumbent cough, troublesome cough, breathing difficulties), indicating a high likelihood of symptom co-occurrence (Table 2).

**Table 1.** Descriptive statistics of LPRS.

Symptoms	$\bar{x}$	s	RSI score
S1	1.434	0.668	1.434 ± 0.668
S2	1.869	1.305	1.869 ± 1.305
S3	1.647	1.086	1.647 ± 1.086
S4	1.251	0.663	1.251 ± 0.663
S5	1.277	0.738	1.277 ± 0.738
S6	1.191	0.625	1.191 ± 0.625
S7	1.386	0.915	1.386 ± 0.915
S8	1.582	1.091	1.582 ± 0.915
S9	1.376	0.766	1.376 ± 0.766

$\bar{x}$ : mean scores of each LPRS which was calculated by dividing the total RSI score with the total number of individuals investigated; *s*: standard deviation; RSI score: $\bar{x}$ ± *s*; S1: Did you have hoarseness or any problem with your voice; S2: Did you have a problem of clearing your throat; S3: Did you have a problem of excess throat mucus or postnasal drip; S4: Did you have difficulty in swallowing food, liquids or pills; S5: Did you cough after eating or lying down; S6: Did you have breathing difficulties or choking episodes; S7: Did you have troublesome or annoying cough; S8: Did you have the sensation of something sticking or a lump in your throat; S9: Did you have heartburn, chest pain, indigestion or stomach acid regurgitation.

**Table 2.** Bivariate correlations analysis among LPRS.

	S1	S2	S3	S4	S5	S6	S7	S8	S9	RSI
S1	1	0.31	0.38	0.39	0.31	0.4	0.36	0.41	0.35	0.61
S2	0.31	1	0.39	0.23	0.27	0.24	0.28	0.32	0.3	0.58
S3	0.38	0.39	1	0.45	0.42	0.34	0.31	0.56	0.41	0.7
S4	0.39	0.23	0.45	1	0.42	0.45	0.33	0.45	0.49	0.63
S5	0.31	0.27	0.42	0.42	1	0.52	0.57	0.45	0.49	0.67
S6	0.4	0.24	0.34	0.45	0.52	1	0.44	0.41	0.57	0.62
S7	0.36	0.28	0.31	0.33	0.57	0.44	1	0.52	0.41	0.67
S8	0.41	0.32	0.56	0.45	0.45	0.41	0.52	1	0.49	0.74
S9	0.35	0.3	0.41	0.49	0.49	0.57	0.41	0.49	1	0.7
RSI	0.61	0.58	0.7	0.63	0.67	0.62	0.67	0.74	0.7	1

S1: Did you have hoarseness or any problem with your voice; S2: Did you have a problem of clearing your throat; S3: Did you have a problem of excess throat mucus or postnasal drip; S4: Did you have difficulty in swallowing food, liquids or pills; S5: Did you cough after eating or lying down; S6: Did you have breathing difficulties or choking episodes; S7: Did you have troublesome or annoying cough; S8: Did you have the sensation of something sticking or a lump in your throat; S9: Did you have heartburn, chest pain, indigestion or stomach acid regurgitation.

### Overview of lifestyle behaviours and dietary habits

**Figure 2.**
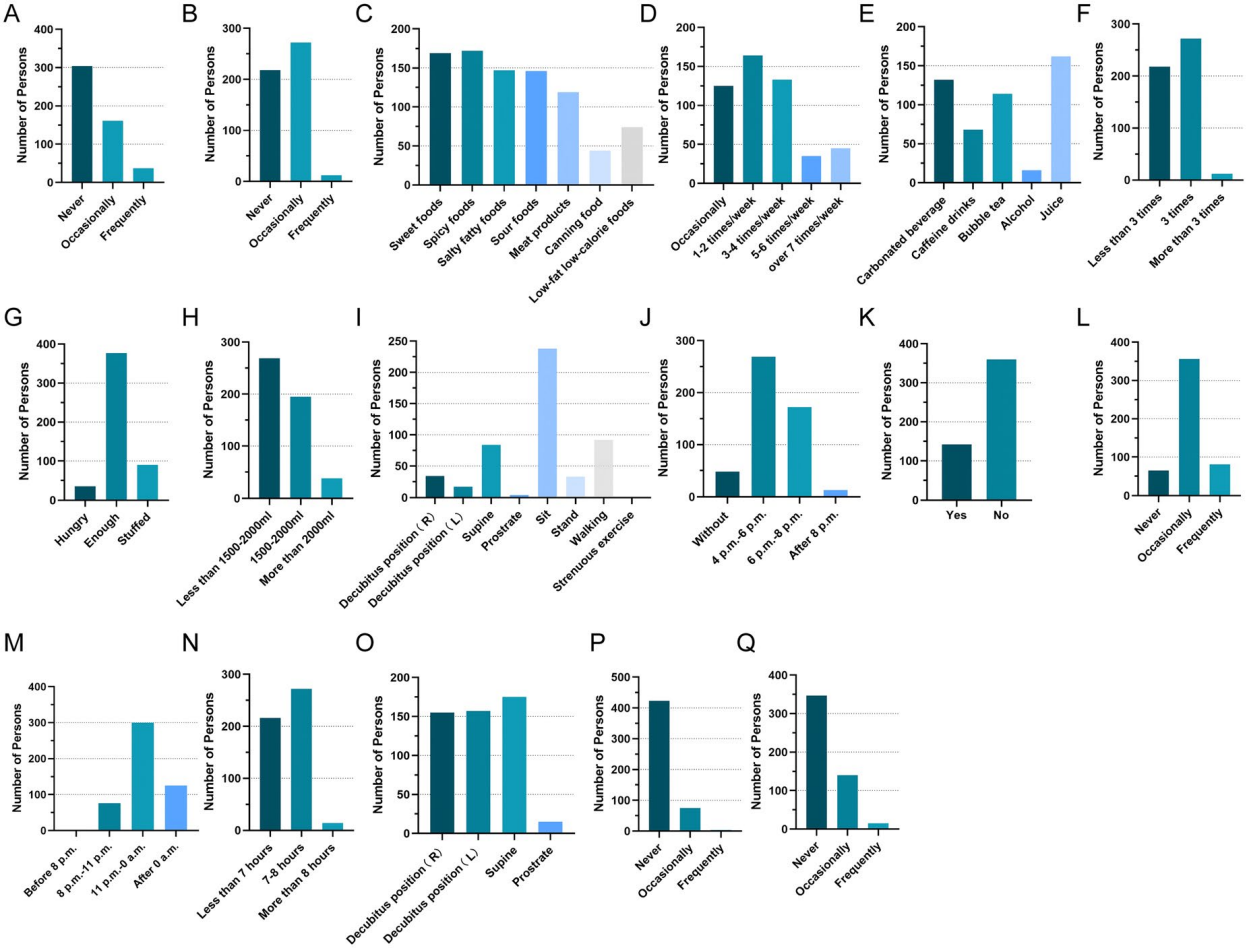
**Lifestyle and dietary habits of college students.** (A) Tobacco exposure. (B) Alcohol intake. (C) Dietary preferences. (D) Intake frequency of dietary preferences. (E) Beverage preferences. (F) Frequency of daily meals. (G) Feeling after each meal. (H) Daily water consumption. (I) Status within 30 min after a meal. (J) Dinner time. (K) Night snack habits. (L) Frequency of exercises. (M) Bed time. (N) Sleeping hours. (O) Sleep postures. (P) Application of PPIs. (Q) Application of NSAIDs. PPIs: proton pump inhibitors; NSAIDs: non-steroidal anti-inflammatory drugs.

Most respondents reported no tobacco exposure (304/502) and only occasional social drinking (272/502) (Figure 2(A) and (B)). In terms of diet, there was a general preference for sweet, spicy, high-salt, high-fat and meat-based foods, with most individuals consuming these foods 1–2 times per week (Figure 2(C) and (D)). Dinner was typically eaten between 4 PM and 6 PM (269/502), and nighttime snacking was not common (360/502) (Figure 2(J) and (K)). Furthermore, this population generally lacked regular physical activity, had insufficient daily water intake and slept less than 8 h per night (Figure 2(E)–(N)). The use of proton pump inhibitors (PPIs) or non-steroidal anti-inflammatory drugs (NSAIDs) was relatively rare (Figure 2(P) and (Q)).

### Key factors associated with LPRS

**Figure 3.**
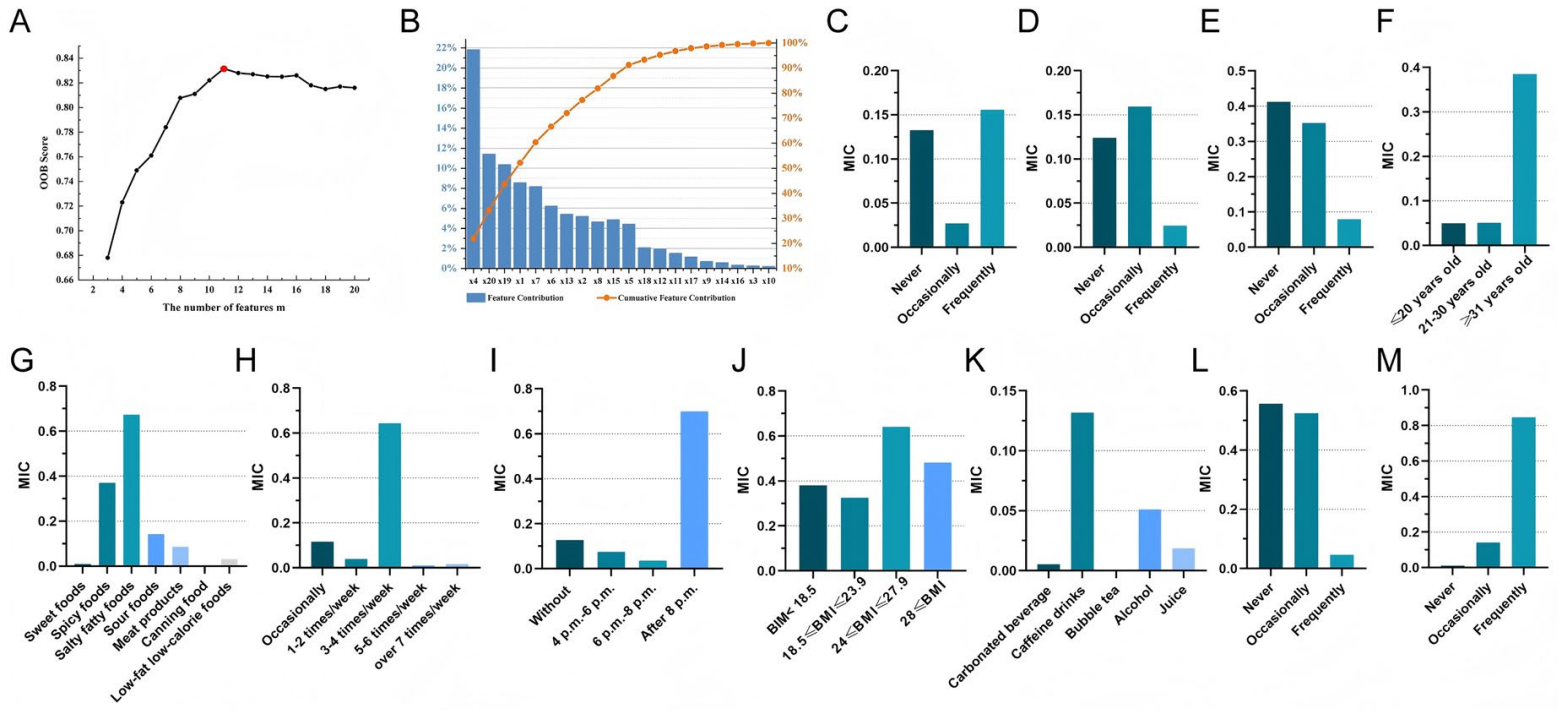
**Effect of lifestyle and dietary habits on LPRS among college students.** (A) OOB scores of features regarding lifestyle and dietary habits of college students. (B) Feature contribution. (C) MIC correlation between tobacco exposure and LPRS. (D) MIC correlation between use of NSAIDs and LPRS. (E) MIC correlation between use of PPIs and LPRS. (F) MIC correlation between age and LPRS. (G) MIC correlation between different dietary preferences and LPRS. (H) MIC correlation between frequency of dietary preferences and LPRS. (I) MIC correlation between dinner time and LPRS. (J) MIC correlation between BMI and LPRS. (K) MIC correlation between beverage preferences and LPRS. (L) MIC correlation between frequency of exercises and LPRS. (M) MIC correlation between alcohol intake and LPRS. LPR: laryngopharyngeal reflux; OOB: Out-of-Bag; MIC: Maximal Information Coefficient; NSAID: non-steroidal anti-inflammatory drug; PPI: proton pump inhibitor; BMI: body mass index.

Based on the Out-of-Bag (OOB) method, we identified 11 features most relevant to LPRS (Figure 3(A) and (B); Supplemental Table 3 and Supplemental Table 4). Among these, tobacco exposure, NSAID use, PPI use, age and dietary preferences ranked among the top five (Supplemental Table 3). Specifically, long-term tobacco exposure, occasional NSAID use, non-use of PPIs, age over 31 years, consumption of high-salt/high-fat/spicy foods more than 3–4 times per week, eating dinner after 8 PM, overweight status, intake of caffeinated beverages, lack of regular exercise and long-term alcohol consumption were all significantly associated with LPRS (Figure 3(C)–(M)).

### Screening performance of the GA-Stacking model

**Figure 4.**
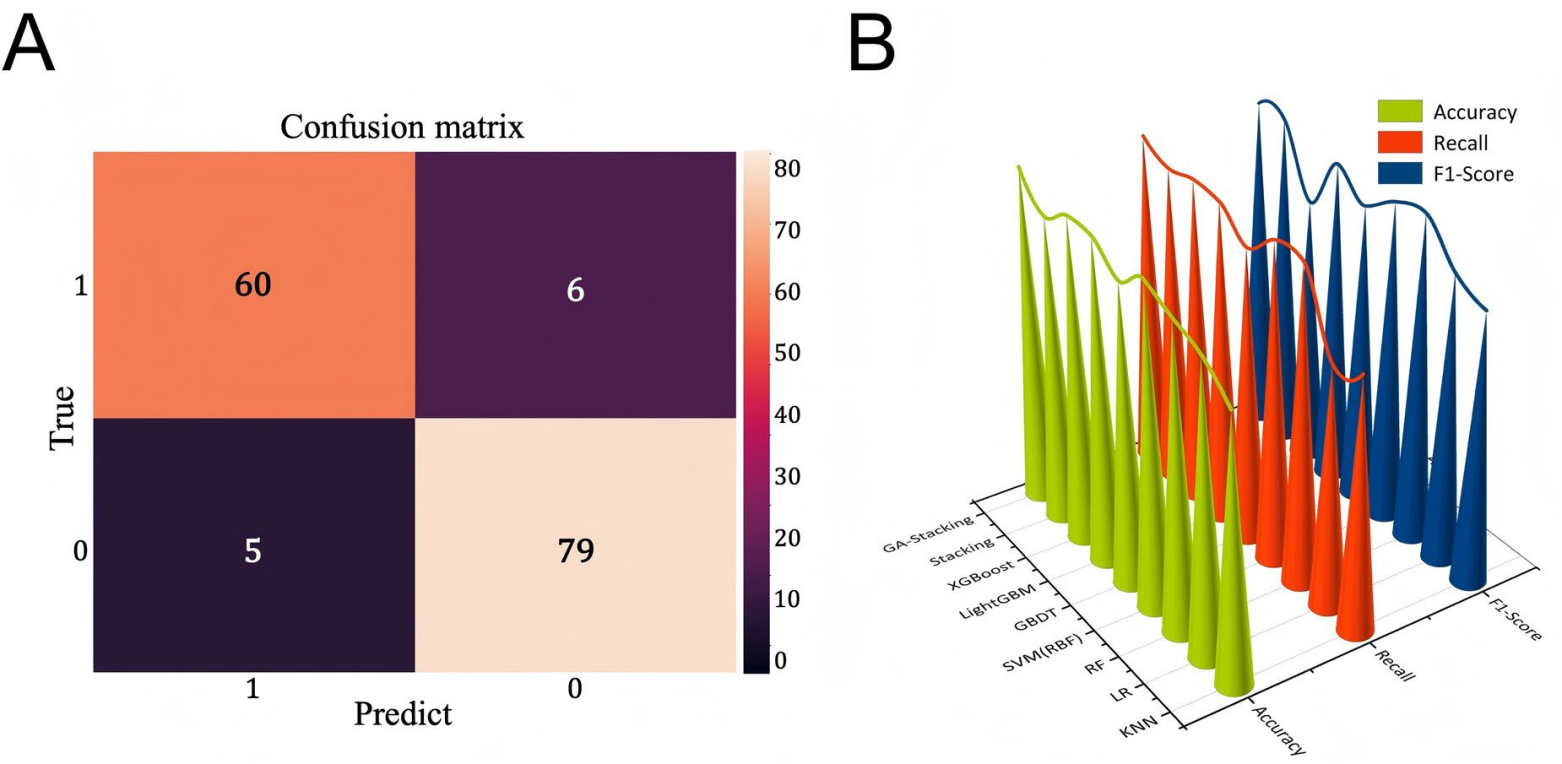
**Performance of GA–Stacking screening model.** (A) GA–SA blending prediction confusion matrix. The orange square stands for TP, the purple square for FP, the dark purple square for FN and the creamy square for TN. (B) The performance of different prediction models. The green, red and blue conical shapes represent the accuracy, recall and F1-scores of the different prediction models, respectively. GA: genetic algorithm; TP: true positive; FP: false positive; TN: true negative; FN: false negative.

On the independent external validation set (*n* = 150), the GA–Stacking model demonstrated high performance, with a recall of 0.909, an accuracy of 0.927 and an area under the receiver operating characteristic curve of 0.96 (95% CI, 0.92–0.98) (Figure 4(A)). In the comparative evaluation, the GA–Stacking model achieved superior accuracy, recall and F1-score compared to the seven individually GA-optimized baseline classifiers and the non-optimized stacking model (Figure 4(B), Supplement Table 5).

## Discussion

Lifestyle behaviours and dietary patterns vary across geographical regions and may influence the development of region-specific health conditions [26]. Promoting health literacy and empowering individuals to make informed decisions about lifestyle behaviours and dietary choices are considered the integral components in the management of chronic conditions [27]. Evidence from GERD research has demonstrated that lifestyle modification and patient education can effectively improve the symptom control [28]. However, comparable recommendations specific to LPR remain limited.

University life represents a critical period during which long-term lifestyle behaviours and dietary habits are established [29–31]. Moreover, this population exhibits high homogeneity in terms of age, educational background, and lifestyle (such as collective accommodation and canteen meals). Therefore, selecting this group as the research object can more clearly reveal the potential associations between lifestyle behaviours, dietary habits and LPRS, with less interference from complex factors such as age, chronic diseases and long-term medication use. Previous studies have indicated that the incidence of LPR in specific populations is significantly higher than that in the general population, and these research findings further support the hypothesis that college students’ lifestyle behaviours and dietary habits may be associated with LPRS [14,15].

The current study using a self-reported questionnaire consisting of 34 items related to physical characteristics, lifestyle behaviours and dietary patterns, along with the RSI (Supplementary materials). The assessment also included the use of NSAIDs and PPIs, as these medications are commonly used and have previously been associated with LPRS presentation. The characteristics of these behaviours and habits are presented in detail (Figure 2). We defined patients with an RSI > 13 as LPRS-positive and analysed the associated lifestyle behaviours and dietary habits accordingly. Given the high-dimensional nature of the data set, traditional feature selection methods were insufficient for identifying the key predictors. Therefore, a composite feature ranking approach was implemented using PCC, MIC, RF, CatBoost and LR analysis, with further refinement based on OOB error from RF. This method yielded 11 primary risk factors associated with LPRS (Figure 3). This study validated several known risk factors for LPR. For example, tobacco exposure (including second-hand smoke) was identified as one of the most influential factors, which is fully consistent with the mechanism documented in numerous studies that smoking exacerbates reflux by impairing oesophageal motility and delaying gastric emptying [32,33]. In addition, occasional use of NSAIDs also showed an association with LPRS, further supporting the hypothesis that they may aggravate reflux symptoms through mucosal injury. Notably, individuals over 31 years of age exhibited higher susceptibility, whereas previous studies suggested that LPRS is less typical in older adults [34]. This may be a result of physiological changes associated with aging or the cumulative effect of long-term behaviours, indicating that this age group may become a new key intervention window.

In terms of diet, our findings provide a more nuanced perspective. Although spicy and sweet foods are the most preferred among college students, high-salt and high-fat foods also show a strong association with LPRS. This is consistent with the known physiological mechanism that they can reduce lower oesophageal sphincter pressure and promote gastric acid secretion [35]. More importantly, we identified the decisive role of ‘frequency’ – consuming these foods more than 3–4 times a week is the threshold for a significant increase in risk. This goes beyond previous studies that only focused on ‘whether to consume’ and provides a quantitative reference for formulating specific dietary recommendations (e.g. ‘no more than twice a week’). Regarding beverages, the effect of alcohol is similar to that of GERD [33], but due to limitations in our methodology, we were unable to accurately quantify caffeine intake, which is an area for improvement in future research.

This study also revealed risk patterns associated with daily rhythms. Eating dinner late (after 8 PM) was correlated with increased symptom frequency, which is likely related to reduce oesophageal clearance during recumbent sleep. An interesting finding regarding body weight is that overweight status showed a stronger association with LPRS than obesity. This suggests that in the college student population, being overweight may be a triggering factor for LPRS, rather than requiring the attainment of clinical obesity criteria [36]. Meanwhile, as a prevalent phenomenon, the association between lack of physical activity and LPRS underscores the importance of regular exercise in maintaining the anti-reflux barrier [37].

**Machine learning** is widely used to estimate disease risk, inform diagnostic processes, guide treatment planning and support clinical decision-making. The increasing availability of high-dimensional medical data has created opportunities for the development of more sophisticated models, as demonstrated in research related to COVID-19 and kidney disease [38,39]. In this study, we developed a novel screening tool for LPRS among college students by using the identified lifestyle and dietary risk factors. The screening tool was based on a stacking ensemble strategy, which integrates multiple machine learning algorithms in a two-layer structure.

Stacking has been reported to outperform traditional methods such as bagging and boosting [40]. A GA was incorporated to optimize the model parameters, following the principle of evolutionary selection to improve predictive accuracy. The GA–Stacking model demonstrated strong performance in terms of accuracy, recall and F1-score (Figure 4(A)). Moreover, the GA–Stacking model outperformed seven conventional classifiers (whose hyperparameters were also optimized using the GA) and a non-optimized stacking model, offering more consistent classification accuracy and greater reliability (Figure 4(B), Supplement Table 5). This demonstrates that the performance gain stems from the synergistic effect of the stacking architecture and the meta-learner’s ability to integrate diverse base model predictions, rather than merely from hyperparameter tuning.

However, this study has several limitations. First, the predictive results of our model are fundamentally based on the RSI, a patient-reported screening tool. Although the threshold of RSI > 13 is widely used to identify individuals with suspected LPR, it must be acknowledged that this threshold lacks perfect specificity. Despite controlling for multiple variables, the data collection method relying on self-reported questionnaires is inevitably subject to recall bias and social desirability bias. Therefore, a high RSI score only indicates the severity of LPRS but cannot confirm a diagnosis of LPR. Similar symptoms from other conditions (such as allergic rhinitis, chronic rhinosinusitis or vocal abuse) may interfere with the results. Consequently, the incidence of LPRS observed in our findings may be higher than the true incidence of LPRS. Thus, the GA–Stacking model predicts the high risk of LPRS rather than etiologically confirmed LPR. It can serve as a tool to identify patients at high risk of LPR symptoms, enabling such individuals to benefit from further clinical evaluation. Future studies need to integrate objective diagnostic measures (such as 24-hour laryngopharyngeal–oesophageal pH monitoring) to validate and optimize the predictive models for confirmed LPR. Additionally, this study is based on a sample of college students in Jilin Province, and great caution should be exercised when directly generalizing its conclusions to other regions, age groups or clinical patient populations. The performance of the model requires validation on independent external datasets.

## Conclusion

The present findings indicated that specific lifestyle behaviours and dietary patterns were associated with LPRS among college students in this exploratory, descriptive analysis. The GA–Stacking model demonstrated strong performance in identifying individuals with a current high burden of LPRS. Future prospective validation in clinical cohorts is essential to confirm these findings and is a necessary step before any screening application can be considered.

We plan to conduct a prospective, multicentre clinical study, consecutively recruiting symptomatic patients from otolaryngology clinics across different regions. All enrolled participants will complete the simplified electronic questionnaire developed in this study and undergo 24-hour laryngopharyngeal–oesophageal pH monitoring, which serves as the objective gold standard for diagnosing LPR. We will use this cohort as an independent external validation set to rigorously evaluate the generalization performance of the GA-Stacking model and further optimize the model to enhance its clinical applicability and robustness.

## Supplementary Material

Supplemental Material

Supplementary materials.docx

Figure S3.jpg

Supplement Tables.xlsx

Figure S1.jpg

Figure S2.jpg

## Data Availability

The data that support the findings of this study are available from the corresponding author, [Dan Yu], upon reasonable request.

## List of abbreviations

LPR: laryngopharyngeal reflux
LPRS: laryngopharyngeal reflux symptom
RSI: Reflux Symptom Index
GERD: gastroesophageal reflux disease
KMO: Kaiser-Meyer-Olykin
BMI: body mass index
SVM: Support Vector Machine
XGBoost: Extreme Gradient Boosting
GBDT: Gradient Boosting Decision Tree
RF: Random Forest
KNN: K-Nearest Neighbour
GA: genetic algorithm
PPI: proton pump inhibitor
NSAID: non-steroidal anti-inflammatory drug
TP: true positive
FP: false positive
TN: true negative
FN: false negative
PCC: Pearson Correlation Coefficient
MIC: Maximal Information Coefficient
RF: Random Forest Algorithm
CatBoost: Gradient Boosting Algorithm
LR: Logistic Regression
OOB: Out-of-Bag.
