# Supplementary material for: Machine learning for screening laryngopharyngeal reflux symptoms in college students: a cross-sectional study
Source: Ann Med. 2026 Jan 5;58(1):2610063. doi: 10.1080/07853890.2025.2610063 (PMC12777997; doi:10.1080/07853890.2025.2610063)
Supplement: Supplemental Material [file IANN_A_2610063_SM6517.docx]

Figure S1. PMRCL fusion feature selection process. PCC: Pearson Correlation Coefficient; MIC: Maximal Information Coefficient; RF: Random Forest Algorithm; LR: Logistic Regression.

Figure S2. The process of GA-Stacking parameter optimization.

Figure S3. Geographic characteristics of the participants. (A) Chinese map presenting the province ranking of participants. (B) The numbers of participants in each ranking province.
