# Supplementary material for: Machine learning for screening laryngopharyngeal reflux symptoms in college students: a cross-sectional study
Source: Ann Med. 2026 Jan 5;58(1):2610063. doi: 10.1080/07853890.2025.2610063 (PMC12777997; doi:10.1080/07853890.2025.2610063)
Supplement: Supplementary materials.docx [file IANN_A_2610063_SM0712.docx]

**The Survey of Relevance of Lifestyle and Dietary Habits for LPR Symptoms in College Students**

This survey is only for the relevance of lifestyle and dietary habits for LPR symptoms in college students. Your records will be remained confidential. Please answer listed questions based on your real condition and experiences in the past month. Thanks for your participations!

1. Age: _______
2. Race: _______
3. Hometown: ________ Province
4. Height: _______ cm
5. Weight: _______ kg
6. Gender:

□ Male

□ Female

1. Do you smoke (Including second-hand smoke)?

□ Never

□ Occasionally

□ Frequently

1. Do you drink?

□ Never

□ Occasionally

□ Frequently

1. Which of the following foods are you favorite? (You could pick one or more than one)

□ Sweet foods

□ Spicy foods

□ Salty fatty foods

□ Sour foods

□ Meat products

□ Canning food

□ Low-fat low-calorie foods

1. What is the frequency of your favorite food intake?

□ Occasionally

□ 1-2 times/week

□ 3-4 times/week

□ 5-6 times/week

□ over 7 times/week

1. Which of the following beverages are you favorite? (You could pick one or more than one)

□ Carbonated beverage

□ Caffeine drinks (coffee and energy drinks)

□ Bubble tea

□ Alcohol

□ Juice

1. How many meals do you have every day?

□ Less than 3 times

□ 3 times

□ More than 3 times

1. How do you feel after each meal？

□ Hungry

□ Enough

□ Stuffed

1. How much water do you drink every day?

□ Less than 1500-2000ml

□ 1500-2000ml

□ More than 2000ml

1. What do you like to do in 30 min after a meal?

□ Right lateral decubitus position

□ Left lateral decubitus position

□ Supine

□ Prostrate

□ Sit

□ Stand

□ Walking

□ Strenuous exercise

1. When do you have your late evening meal?

□ Without a late evening meal

□ 4 p.m.-6 p.m.

□ 6 p.m.-8 p.m.

□ After 8 p.m.

1. Do you eat snacks at night time? (Within two hours before going to bed)

□ Yes

□ No

1. What is the frequency of your exercise?

□ Never

□ Occasionally

□ Frequently

1. What time do you usually fall asleep?

□ Before 8 p.m.

□ 8 p.m.-11 p.m.

□ 11 p.m.-0 a.m.

□ After 0 a.m.

1. How long do you sleep?

□ Less than 7 hours

□ 7-8 hours

□ More than 8 hours

1. What is your most common sleep posture?

□ Left lateral decubitus position

□ Right lateral decubitus position

□ Supine

□ Prostrate

1. Do you have a history of PPIs therapy?

□ Never

□ Occasionally

□ Frequently

1. Do you have a history of NSAIDs therapy?

□ Never

□ Occasionally

□ Frequently

1. Did you have a hoarseness or a problem with your voice?

□ Never

□ Occasionally

□ 1-2 times/week

□ 3-4 times/week

□ 5-6 times/week

□ Everyday

1. Did you have a problem of clearing your throat?

□ Never

□ Occasionally

□ 1-2 times/week

□ 3-4 times/week

□ 5-6 times/week

□ Everyday

1. Did you have a problem of excess throat mucus or postnasal drip?

□ Never

□ Occasionally

□ 1-2 times/week

□ 3-4 times/week

□ 5-6 times/week

□ Everyday

1. Did you have a difficulty in swallowing food, liquids, or pills?

□ Never

□ Occasionally

□ 1-2 times/week

□ 3-4 times/week

□ 5-6 times/week

□ Everyday

1. Did you have a cough after you ate or after lying down?

□ Never

□ Occasionally

□ 1-2 times/week

□ 3-4 times/week

□ 5-6 times/week

□ Everyday

1. Did you have breathing difficulties or choking episodes?

□ Never

□ Occasionally

□ 1-2 times/week

□ 3-4 times/week

□ 5-6 times/week

□ Everyday

1. Did you have troublesome or annoying cough?

□ Never

□ Occasionally

□ 1-2 times/week

□ 3-4 times/week

□ 5-6 times/week

□ Everyday

1. Did you have sensations of something sticking in your throat or a lump in your throat?

□ Never

□ Occasionally

□ 1-2 times/week

□ 3-4 times/week

□ 5-6 times/week

□ Everyday

32. Did you have heartburn, chest pain, indigestion, or stomach acid coming up?

□ Never

□ Occasionally

□ 1-2 times/week

□ 3-4 times/week

□ 5-6 times/week

□ Everyday

1. Which season did you have symptom18-26?

□ Spring

□ Summer

□ Autumn

□ Winter

1. How about the duration of symptom18-26

□ Fluctuating

□ Persistent (□ a. without aggravation: □ ≤3 months，□ >3 months;

□ b. increasing progressively：□ ≤3 months，□ >3 months)
