# Supplementary figures and images for: Machine learning for screening laryngopharyngeal reflux symptoms in college students: a cross-sectional study
Source: Ann Med. 2026 Jan 5;58(1):2610063. doi: 10.1080/07853890.2025.2610063 (PMC12777997; doi:10.1080/07853890.2025.2610063)

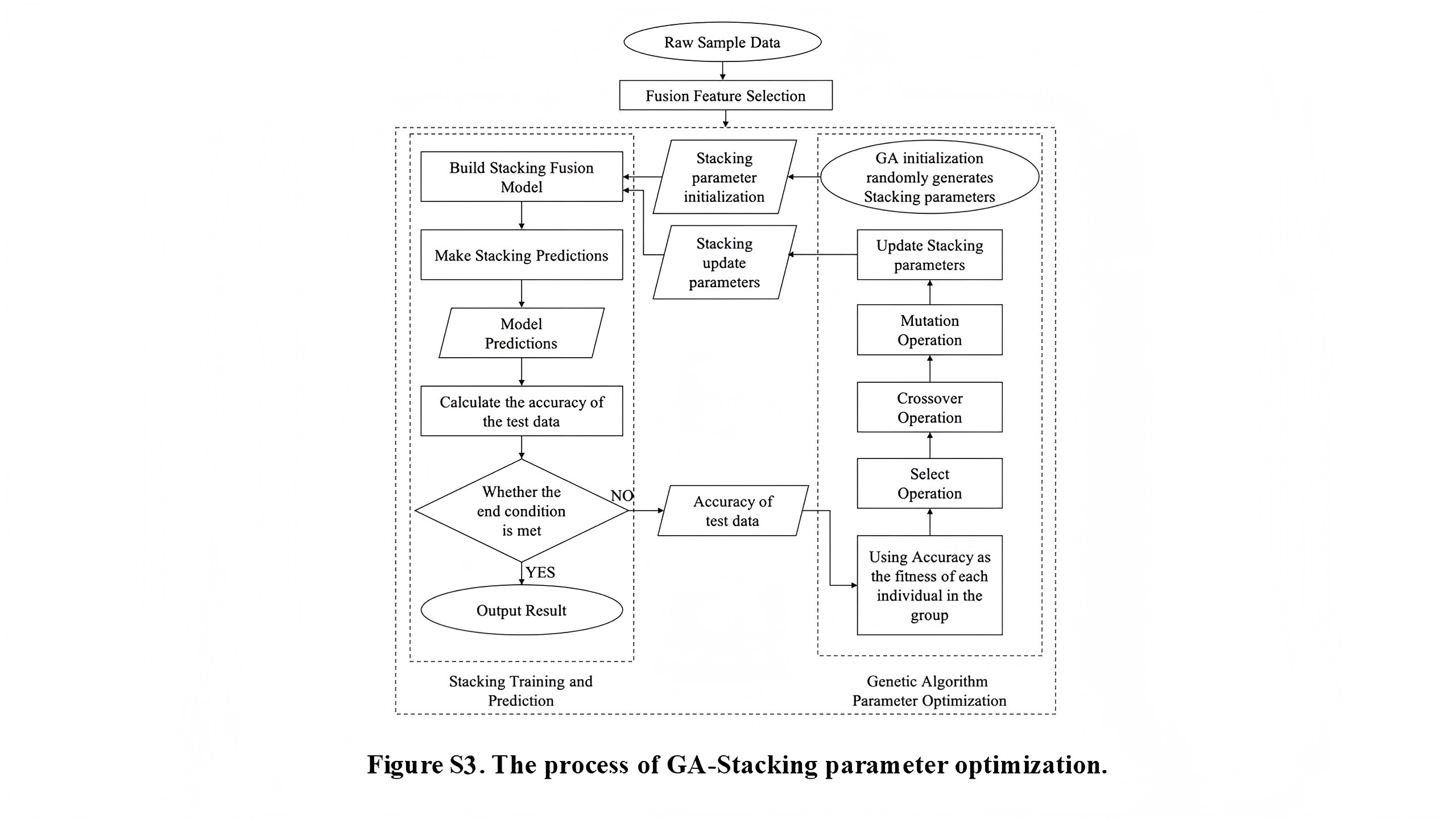

Supplement: Figure S3.jpg [file IANN_A_2610063_SM0711.jpg]

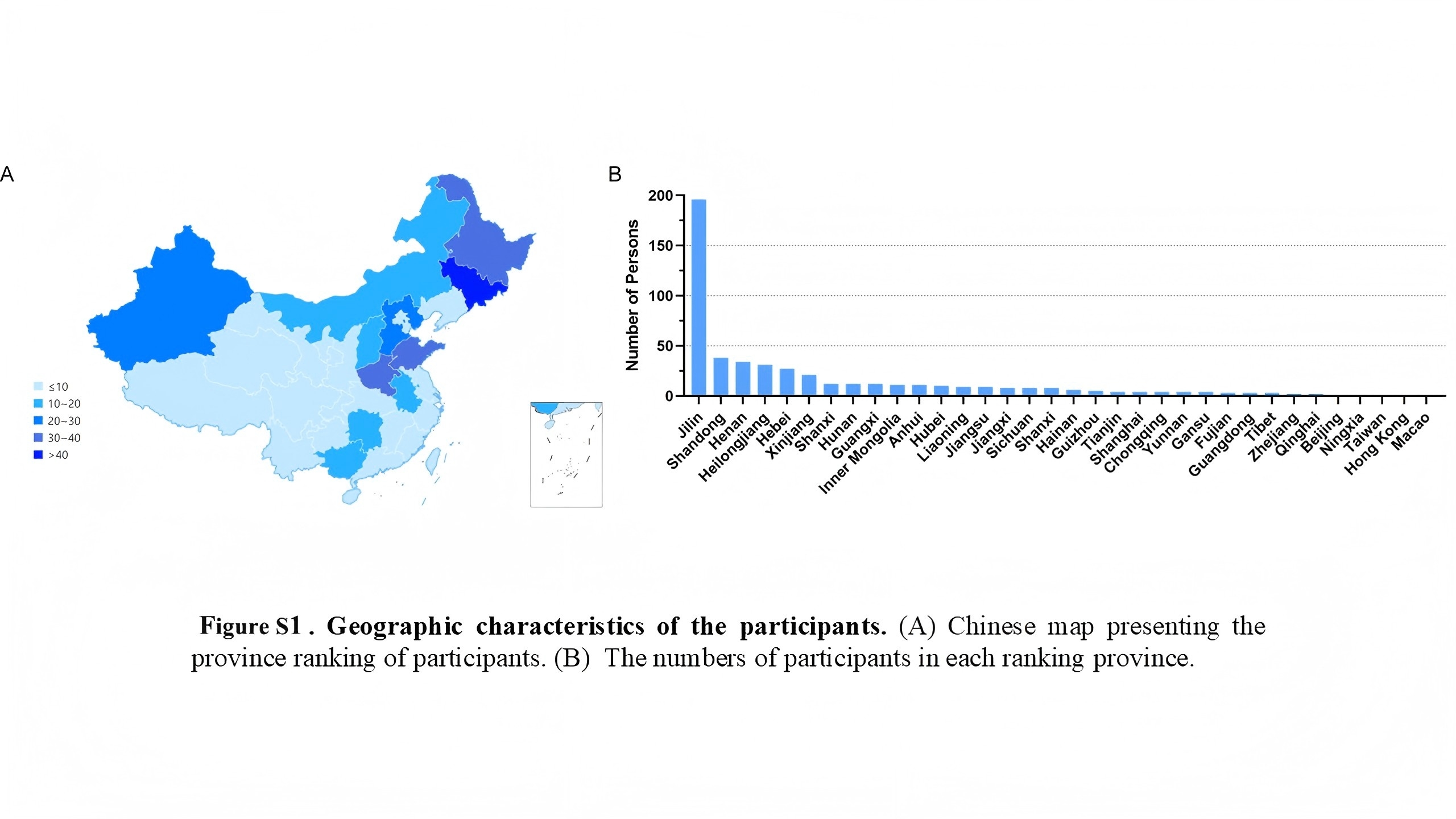

Supplement: Figure S1.jpg [file IANN_A_2610063_SM0709.jpg]

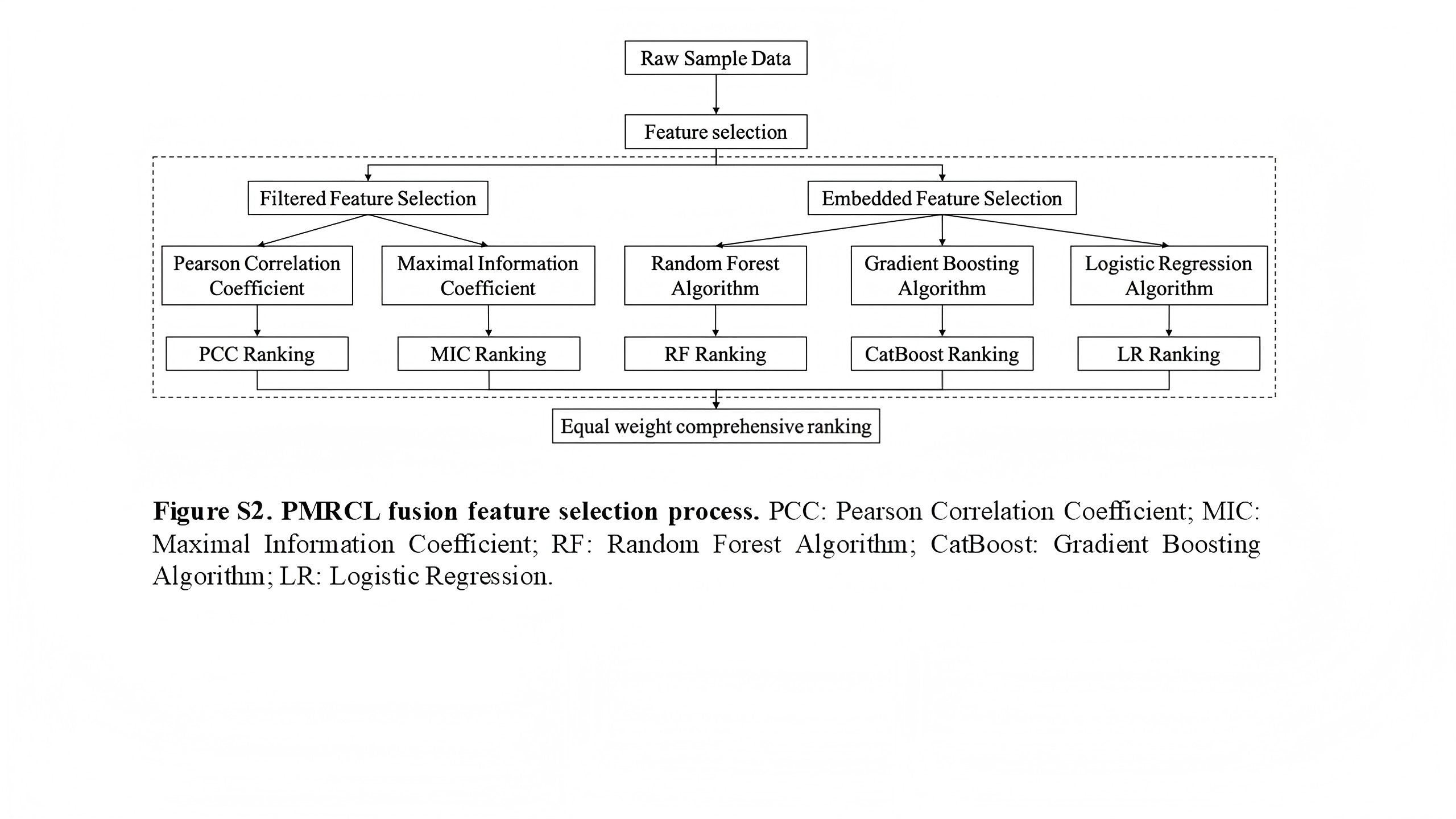

Supplement: Figure S2.jpg [file IANN_A_2610063_SM0708.jpg]
